# Identification of empagliflozin-related hub genes in atherosclerosis and their correlations with immune infiltration: Network pharmacology and bioinformatics analyses

**DOI:** 10.1371/journal.pone.0339956

**Published:** 2026-01-16

**Authors:** Yicheng Rong, Xinyu Liu, Yuanyuan Sun, Le Yang, Liming Chen

**Affiliations:** 1 School of Life Sciences, Nanjing University, Nanjing, China; 2 Department of Cardiology, Shandong Provincial Hospital Affiliated to Shandong First Medical University, Jinan, Shandong, China; 3 Department of Geriatric Cardiology, Shandong Provincial Hospital Affiliated to Shandong First Medical University, Jinan, Shandong, China; Chiba University, JAPAN

## Abstract

**Background:**

Atherosclerosis (AS) is by far the most frequent underlying cause of atherosclerotic cardiovascular disease. Recently, sodium-glucose cotransporter 2 (SGLT2) inhibitors stand out for their anti-atherosclerotic effects. The present study was conducted to explore the potential genetic and molecular mechanisms of empagliflozin, a selective SGLT2 inhibitor, in preventing AS, and the correlation of empagliflozin-related hub genes with immune cells.

**Methods:**

In our study, pharmacology platforms were accessed to identify the empagliflozin-related genes (ERGs). AS datasets GSE100927 and GSE43292 were downloaded from the public GEO database to identify AS-differentially expressed genes (AS-DEGs). Furthermore, the empagliflozin-related DEGs (ERDEGs) were obtained by intersecting AS-DEGs and ERGs. ERDEGs were further analyzed for Gene ontology (GO) and Kyoto Encyclopedia of Genes and Genomes (KEGG) pathway enrichment analyses. The protein-protein interaction (PPI) network was constructed to screen for hub genes, which were subjected to regulatory network construction, ROC curve plotting, as well as validation analysis, correlation and Friends analysis, and CIBERSORT and ssGSEA analysis.

**Results:**

A total of 33 genes were identified as ERDEGs, among which the empagliflozin-related hub genes were identified to be IL1B, EGFR, ERBB2, JAK2, SYK, and LGALS3 in AS. Furthermore, in terms of the characteristics of immune cells, there were significant correlations between ERBB2 and CD8 + T cells, IL1B and resting mast cells, LGALS3 and eosinophils, as well as JAK2 and CD56dim NK.

**Conclusion:**

Our network pharmacology and bioinformatics analyses provide a comprehensive understanding of the potential mechanisms of empagliflozin in AS at the genomic level, with the discovery of significant correlations between the screened hub genes and various immune cell subsets.

## 1. Introduction

Atherosclerosis (AS) is a chronic large and medium-sized artery-involved inflammatory disease that may induce atherosclerotic cardiovascular disease (ASCVD) involvingischemic heart disease, stroke, and peripheral vascular disease. AS has affected humans for millennia [1,2]. Currently, the primary anti-atherosclerotic therapies included cardioprevention guideline-recommended treatments and effective lipid-lowering medications (e.g., statins, ezetimibe, and PCSK9 inhibitors), either individually or in combination. However, ASCVD continues to be a leading cause of death globally, responsible for approximately 17.6 million deaths annually, with its incidence on the rise. It imposes a heavy disease burden clinically, affecting quality of life, and escalating healthcare costs [3–5]. Noticeably, the remaining risk for major adverse cardiovascular events may be inflammatory in nature in the context of controlled low-density lipoprotein-cholesterol (LDL-C) [1]. Therefore, in order to improve prognosis and achieve better survival for ASCVD patients, novel anti-atherosclerotic treatments can be established preferentially by modulating inflammatory pathway/mechanism.

Serving as a new class of anti-diabetic therapy, SGLT2 inhibitors have been confirmed to possess significant extra-glycaemic benefits in controlling cardiovascular risk factors [6]. In particular, the landmark EMPA-REG OUTCOME study, for the first time, offered convincing evidence that empagliflozin could reduce the total burden of cardiovascular complications in patients with type 2 diabetes and ASCVD [7]. Moreover, several preclinical studies in animal models have further supported the anti-atherosclerotic effect of empagliflozin, with ongoing research exploring potential protective mechanisms, such as suppressing inflammation and sympathetic activity, decreasing vasoconstrictive eicosanoids and inflammation in the vasculature and perivascular adipose tissue, reducing macrophage infiltration in atherosclerotic plaques, etc [8–11]. However, there is currently a lack of comprehensive research analyzing the genetic and molecular mechanisms underlying SGLT2 inhibitors in AS, as well as investigations into their interactions within the human genome.

Network pharmacology, proposed by Hopkins et al. in 2007, is a promising tool to establish a network-centric perspective of drug action through the integration of bioinformatics and cheminformatics [12–14]. Currently, in the era of big data, network pharmacology has been transitioned significantly, supported by a wealth of biological data generated from high-throughput technologies such as sequencing [14]. The practicality network pharmacology-based genetic and molecular research has been further enhanced owing to the public availability of system pharmacology platforms and bioinformatic databases [15].

**Fig 1:**
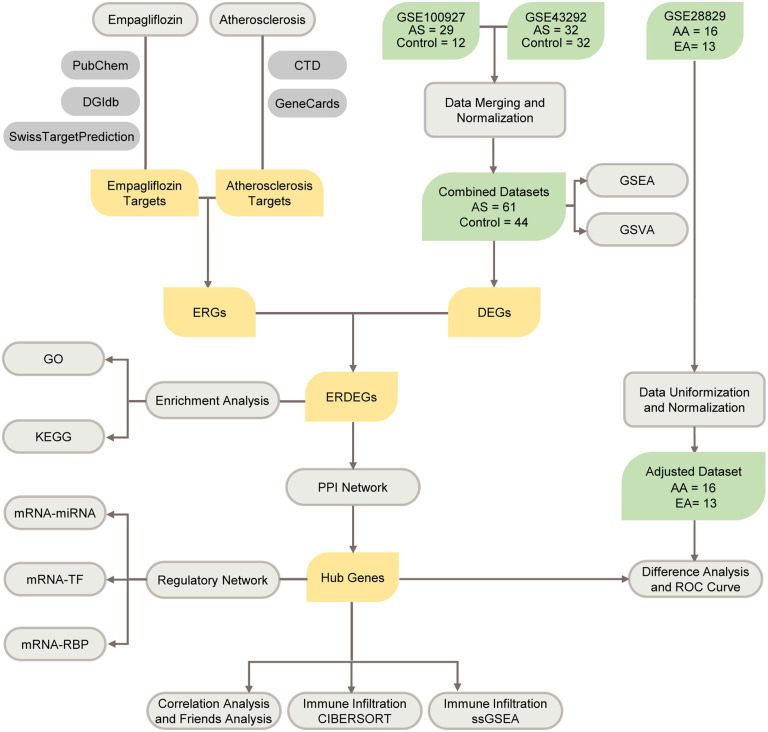
Flow Chart for the Comprehensive Analysis of ERDEGs. AS, Atherosclerosis; AA, Advanced Atherosclerotic Plaque Samples; EA, Early Atherosclerotic Plaque Samples; DEGs, Differentially Expressed Genes; ERGs, Empagliflozin-Related Genes; ERDEGs, Empagliflozin-Related Differentially Expressed Genes; GSEA, Gene Set Enrichment Analysis; GSVA, Gene Set Variation Analysis; GO, Gene Ontology; KEGG, Kyoto Encyclopedia of Genes and Genomes; PPI Network, Protein-Protein Interaction Network; TF, Transcription Factor; RBP, RNA-Binding Protein; ROC Curve, Receiver Operating Characteristic Curve; ssGSEA, single-sample Gene-Set Enrichment Analysis.

In this study, cross-public datasets were accessed to extensively screen potential empagliflozin-related hub genes in AS. Our analysis included target identification, protein-protein interaction network analysis, regulatory network analysis, and hub gene analysis. Unlike previous studies that focused on single aspects of SGLT2 inhibitor mechanisms, our approach systematically combines target prediction, differential gene expression analysis, protein-protein interaction networks, and immune cell correlation analysis to provide a holistic understanding of empagliflozin’s anti-atherosclerotic effects at the genomic level. A comprehensive analysis of hub genes and immune cell characteristics was also adopted given the interdependence between AS and inflammation. Our study aimed to uncover the common molecular regulatory networks of empagliflozi in AS (Fig 1). Findings in this study are expected to provide valuable insights for anti-atherosclerotic mechanism research, as well as novel insights and intervention targets for clinical anti-atherosclerotic drug development.

## 2. Materials and methods

### 2.1. Prediction of targets

Empagliflozin targets were searched and acquired, with “Empagliflozin” as the search term, from PubChem (https://pubchem.ncbi.nlm.nih.gov), [16] SwissTargetPrediction (http://swisstargetprediction.ch/), [17] and DGIdb (https://dgidb.org/) [18]. The online platform-sourced targets were further integrated and visualized using Cytoscape [19]. Potential disease targets were identified from the CTD (https://ctdbase.org/) and GeneCards (https://www.genecards.org/) databases, using “Atherosclerosis” as the key keyword, followed by the intersection of the results [20]. Finally, empagliflozin targets and AS targets were intersected to obtain the ERGs.

### 2.2. Data acquisition and preparation

Using the R package “GEOquery”, AS datasets GSE100927, [21] GSE43292, [22] and GSE28829 [21] were downloaded from the GEO database (https://www.ncbi.nlm.nih.gov/geo/) [23,24]. All samples in these datasets were sourced from Homo sapiens Carotid Arteries. The chip platforms for each dataset were GPL17077, GPL6244, and GPL570, respectively (Table 1). Specifically, GSE100927 and GSE43292 were integrated to create a combined test set (hereinafter referred to as the combined dataset), after merging using the R package “sva” [25] to eliminate batch effects; while GSE28829 served as the validation set. Noticeably, prior to statistical analysis, the R package “limma” was used to conduct background correction and normalization procedures for the combined dataset [26]. In addition, to assess the efficacy of the process, PCA was employed to visualize the merged matrix both before and after batch effect removal [27].

**Table 1:** GEO microarray chip information.

	GSE100927	GSE43292	GSE28829
Platform	GPL17077	GPL6244	GPL570
Experiment type	Expression profiling by array	Expression profiling by array	Expression profiling by array
Species	Homo sapiens	Homo sapiens	Homo sapiens
Tissue	Carotid Arteries	Carotid Arteries	Carotid Arteries
Samples in AS group	29	32	16 (AA)
Samples in Control group	12	32	13 (EA)
Reference	PMID: 36316672	PMID: 31487691	PMID: 36316672

GEO, Gene Expression Omnibus; AS, Atherosclerosis; AA, Advanced Atherosclerotic Plaque Samples; EA, Early Atherosclerotic Plaque Samples.

### 2.3. Identification of ERDEGs

The samples in the combined dataset were divided into AS and control groups for inter-group differential gene analysis using the R package “limma”. AS-DEGs were identified for those with |log FC| ≥ 0.25, and adjusted p(adj. p)<0.05. The |logFC| ≥ 0.25 was chosen based on biological relevance rather than statistical optimization alone. This relatively lenient threshold was selected to capture genes with modest but potentially meaningful biological changes, as network pharmacology studies often identify therapeutic targets through cumulative effects of multiple genes with moderate expression changes rather than focusing solely on highly differentially expressed genes. The |logFC| ≥ 0.25 threshold was selected based on the specific requirements of network pharmacology analysis, where small fold changes may still be biologically meaningful for multi-target drug mechanisms. Lower thresholds are appropriate for pathway analysis when balanced with stringent statistical significance criteria, and our integration with p(adj. p)<0.05 ensures reliable identification of differentially expressed genes while maintaining comprehensive coverage for pathway enrichment analysis. P-values were adjusted using Benjamini-Hochberg (BH) algorithm and a significant DEGs volcano map was drawn using the R package “ggplot2”. Subsequently, the ERDEGs were obtained by intersecting AS-DEGs and ERGs, which were visualized using the Venn diagram. Additionally, this study continued to analyze the expression differences of ERDEGs between different sample groups in the combined datase, and visualized corresponding results using the R package “pheatmap”.

### 2.4. PPI network construction and hub gene screening

This study further constructed the PPI network, using the Cytoscape software and the Search Tool for the Retrieval of Interacting Genes (STRING), to enhance the comprehension of the interactions among ERDEGs [28]. A statistically significant interaction was confirmed whe the combined score was > 0.4. Using the CytoHubba plug-in in Cytoscape, [29] each gene was scored based on the maximal clique centrality (MCC), maximum neighborhood component (MNC), degree, EPC, and Closeness algorithms [30]. Finally, the top-10 genes in each algorithm were considered hub genes. The intersection of these hub genes at the intersection of the five algorithms, designated as empagliflozin-related hub genes for AS was presented in a Venn diagram.

### 2.5. GO and Kyoto KEGG pathway enrichment analysis of ERDEGs

The identified ERDEGs were subjected to GO and KEGG pathway enrichment analyses using the R package “clusterProfiler” [31–33]. The BH algorithm was used for the correction of the p value. Enrichment was considered significant at adj. p < 0.05 and FDR value (q value) < 0.25. In addition, a drug-target-pathway network diagram was created in Cytoscape, based on the drug-target interaction network of empagliflozin, ERDEGs, GO terms, and enriched KEGG pathways.

### 2.6. Gene set enrichment analysis (GSEA)

GSEA assessed whether genes from a predefined set showed a non-random distribution pattern within a gene list ranked by their correlation with the phenotype, thereby indicating the set’s contribution to the phenotype [34]. Following the ranking of genes in the combined dataset from highest to lowest based on logFC values, the GSEA was performed using the R package “clusterProfiler”. The parameters for GSEA were: seed, 2,020; number of calculations, 5,000; minimum gene set size, 10; and maximum gene set size, 500. The gene set c2.all.v2022.1.Hs.symbols.gmt[Curated/Pathway](6449) in the Molecular Signatures Database (MSigDB) were subjected to GSEA [35]. Significant enrichment was determined based on the criteria of adj.P < 0.05 (adjusted using the BH algorithm) and FDR < 0.25.

### 2.7. Gene set variation analysis (GSVA)

In our study, in order to explore the different pathways enriched between the AS and control groups for all genes in the combined dataset, GSVA, a non-parametric unsupervised analysis, [36] was performed using MSigDB-derived hallmark gene set (h.all.v7.4.symbols.gmt) [35]. P-values were adjusted using the BH algorithm and the significance threshold was set at adj. p < 0.05.

### 2.8. Regulatory network of hub genes

Corresponding regulatory networks were constructed to further understand the regulatory mechanism of hub genes. MicroRNAs (miRNAs) are small functional ncRNA molecules that are involved in a wide range of biological processes [37]. Individual miRNAs typically regulate numerous target mRNAs, while a single mRNA transcript is often targeted by multiple miRNAs, forming complex regulatory networks [38]. After obtaining miRNAs associated with hub genes from the miRDB database (https://mirdb.org/), this study continued to clarify the relationship between empagliflozin-related hub genes and miRNA [39]. Given the role of transcription factors (TFs) in controlling gene dynamics and expression, TFs regulating the transcription of empagliflozin-related hub genes potentially were identified in CHIPBase (http://rna.sysu.edu.cn/chipbase/) [40] and the Human Transcription Factors Target (hTFtarget) database (https://bio.tools/hTFtarget) [41] to further illustrate the potential regulation network for these hub genes. RNA-binding Proteins (RBP), a vital player in post-transcriptional gene regulation, are involved in RNA splicing, maturation, transport, stability, degradation, and translation [42]. Therefor, the starBase v3.0 was searched to identify the targeting relationship between RBPs and empagliflozin-related hub genes [43]. Eventually, the Cytoscape software was used to visualize regulatory networks.

### 2.9. Correlation analysis among empagliflozin-related hub genes

To explore pairwise gene expression correlation for empagliflozin-related hub genes, the Spearman correlation analysis was performed and the results were visualized through correlation chord diagrams using the R packages “igraph” and “ggraph”. Additionally, the Friends analysis was also utilized to analyze the functional similarity of the six genes identified in the aforementioned analyses using the R package “GOSemSim” [44].

### 2.10. The ROC curve analysis of the correlation between hub genes expression and AS clinical characteristics

The ROC curve was plotted, using R package pROC, and analyzed to assess the accuracy of the hub gene as a marker for AS and control groups in the combined dataset. Simultaneously, we also generated the comparison chart based on the expression levels of hub genes. Meanwhile, the AUC was calculated to assess the diagnostic impact of hub gene expression on AS occurrence. The hub genes with AUC > 0.7 were deemed useful for disease diagnosis.

### 2.11. Validation of hub genes

To determine the clinical characteristics of AS, the GSE28829 dataset, consisting of 13 EA and 16 AA, was used for to further verify the expression differences of empagliflozin-related hub genes. P-value < 0.05 was considered statistically significant.

### 2.12. Immune cell characteristics and hub gene correlation analysis

Considering the significance of inflammation and immune cells in AS, the CIBERSORT algorithm (https://cibersort.stanford.edu/index.php) and ssGSEA were applied to evaluate the proportions of immune cells along with immune-related functions between AS and control groups [45,46].

Using the CIBERSORT algorithm, the proportions of the 22 types of immune cells were determined based on samples from the combined dataset, with the results visualized by bar charts. Subsequently, the R package “ggplot2” was utilized to compare the levels of the 22 types of immune cells between AS and controls, selecting results showing statistical significance (P < 0.05) for further analysis.

Furthermore, the ssGSEA algorithm was utilized to evaluate the infiltration abundance of 28 different immune cell types within each individual samples. Initially, the infiltrating immune cell types were categorized, which included activated CD8 T cell, activate dendritic cell, gamma delta T cell, natural killer cell, regulatory T cell, etc. Subsequently, immune cell subsets with significant differences between groups were filtered for further analysis. The correlation between immune cell subsets, calculated by Spearman algorithm, was visualized in a correlation heatmap using the R package “pheatmap”. Finally, the correlation between hub genes and immune cell subsets was visualized using a correlation bubble plot generated by the R package “ggplot2”.

### 2.13. Statistical analysis

All statistical analyses in our study were conducted with R software (version 4.2.2), unless otherwise specified, with a two-sided p-value < 0.05 considered statistically significant. Inter-group comparisons of continuous variables with normal distribution was estimated by a Student’s t-test; while differences in non-normally distributed variables across groups were analyzed by the Mann-Whitney U test (i.e., the Wilcoxon rank-sum test). Comparisons between three or more groups were performed by Kruskal-Wallis tests.

### 2.14. Ethics statement

No further institutional review board approval is needed for this study as it was conducted based on secondary analyses of publicly available and de-identified data.

## Results

### 3.1. Prediction of targets

**Fig 2:**
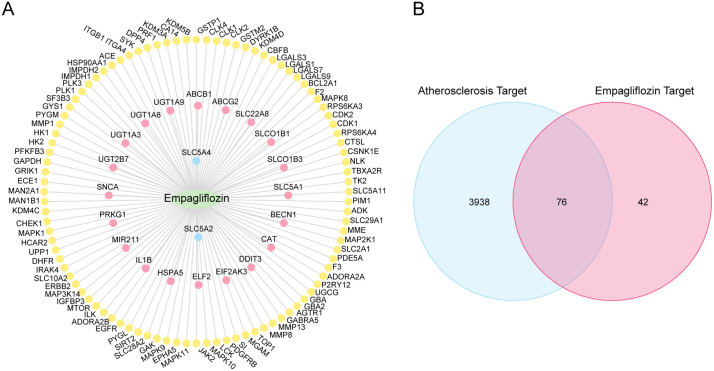
Empagliflozin and Atherosclerosis Targets Interaction Analysis. **A.** empagliflozin-atherosclerosis targets interaction network. The green oval is empagliflozin, and the blue circles are the predicted DGIdb targets; Pink circles are PubChem predicted targets; Yellow circles are the predicted targets from SwissTargetPrediction. **B.** Venn diagram of targets of the drug empagliflozin and disease atherosclerosis. E, Empagliflozin; ERGs, Empagliflozin-Related Genes; AS, Atherosclerosis; ASRGs, atherosclerosis-related Genes.

With access to PubChem, SwissTargetPrediction online platform, and DGIdp, we acquired 22, 100, and 2 empagliflozin targets respectively. After integration, 118 empagliflozin targets were identified, which were visualized by Cytoscape in Fig 2A. Searching in CTD and GeneCards resulted in 36,768 and 4,043 AS targets, with 4,014 targets yielded after merging. Finally, 76 ERGs were obtained by intersecting empagliflozin and AS targets (Fig 2B). Supplementary S1 Table shows the results of the preliminary screening.

### 3.2. Data acquisition and preparation

**Fig 3:**
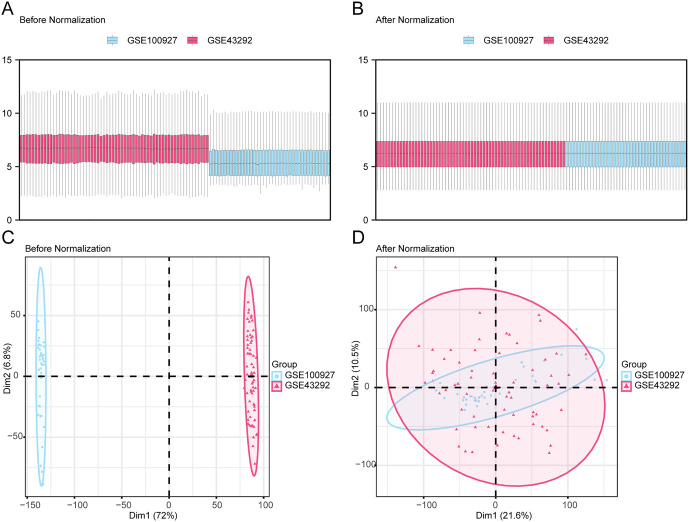
Batch Effects Removal of GSE100927 and GSE43292. **A.** Boxplot of combined datasets distribution before batch removal. **B.** Boxplot of combined dataset distribution post batch removal. **C.** PCA plot of the datasets before debatching. **D.** PCA map of the combined dataset after batch processing. PCA, Principal Component Analysis; AS, Atherosclerosis. The AS dataset GSE100927 is shown in blue, and the AS dataset GSE43292 is shown in pink.

For data acquisition, we merged and normalized GSE100927 (9 AS cases and 12 controls) and GSE43292 (32 AS cases and 32 controls) datasets. A total of 61 AS cases and 44 controls were included in the test set. As revealed in the distribution boxplots and PCA plots, batch processing resulted in an effective elimination of the batch effect of samples in the combined datasets (Fig 3). In addition, the validation set GSE28829 consisted of 16 cases of advanced-stage atherosclerotic plaque (AA) and 13 cases of early-stage atherosclerotic plaques (EA).

### 3.3. Identification of ERDEGs

**Fig 4:**
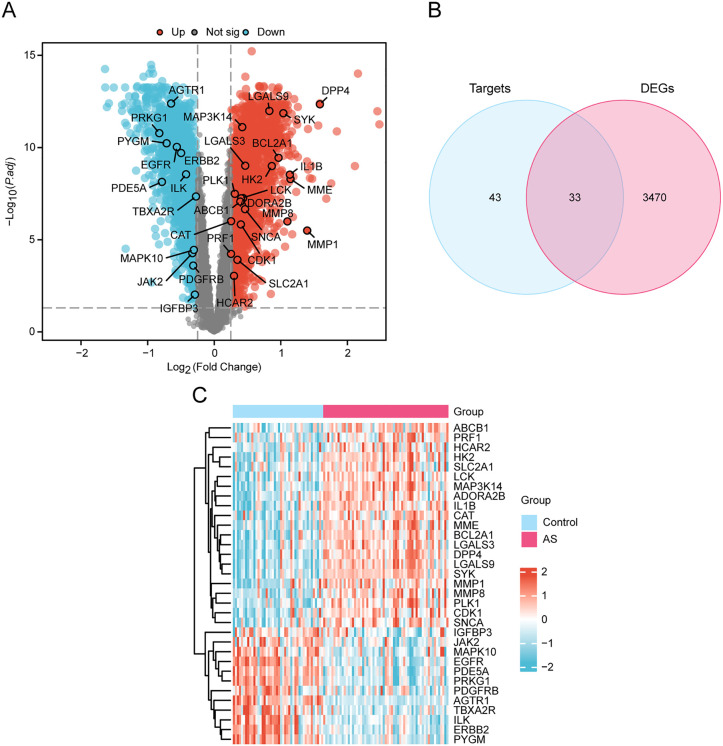
Differential Gene Expression Analysis. **A.** Volcano plot of differentially expressed gene analysis of AS and Control in the combined dataset. The x-axis represents log2 fold change, y-axis represents -log10(adjusted p-value). Red dots indicate significantly upregulated genes (logFC > 0.25, adj.p < 0.05), blue dots indicate significantly downregulated genes (logFC < −0.25, adj.p < 0.05), and gray dots represent non-significant genes. **B.** Venn plot of AS-DEGs and ERGs in the combined datasets. **C.** Heat map of ERDEGs in the combined datasets. AS, Atherosclerosis; DEGs, Differentially Expressed Genes; ERGs, Empagliflozin-Related Genes; ERDEGs, Empagliflozin-Related Differentially Expressed Genes. Pink is AS and blue is Control. Red represents high expression and blue represents low expression in the heat map.

Based on |logFC| > 0.25 and adj.p < 0.05, the combined dataset included 3,503 AS-DEGs. Among these, there were 1,820 up-regulated genes (logFC > 0.25 and adj.p < 0.05) and 1,683 down-regulated genes (logFC < −0.25 and adj.p < 0.05). Differential analysis of these dataset generated a volcano plot, as shown in Fig 4A. After overlapping between all AS-DEGs and the 76 ERGs, 33 genes were identified as ERDEGs (Fig 4B). These genes included ABCB1, ADORA2B, AGTR1, BCL2A1, CAT, CDK1, DPP4, EGFR, ERBB2, HCAR2, HK2, IGFBP3, IL1B, ILK, JAK2, LCK, LGALS3, LGALS9, MAP3K14, MAPK10, MME, MMP1, MMP8, PDE5A, PDGFRB, PLK1, PRF1, PRKG1, PYGM, SLC2A1, SNCA, SYK, and TBXA2R.

### 3.4. PPI network construction and hub gene screening

**Fig 5:**
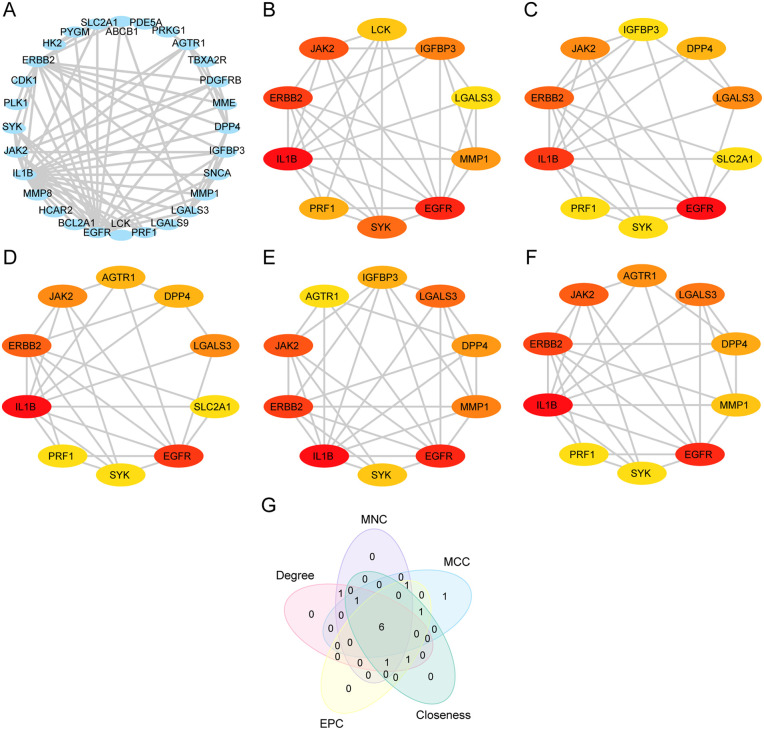
PPI Network and Hub Genes Analysis. **A.** PPI Network of ERDEGs calculated by the STRING database. B-F. PPI Network of TOP10 ERDEGs calculated by 5 algorithms of CytoHubba plug-in, including MCC **(B)**, MNC **(C)**, Degree **(D)**, EPC (E) and Closeness **(F)**. **G.** Venn diagram of the top 10 ERDEGs identified by five algorithms of the CytoHubba plugin. PPI Network, Protein-protein Interaction Network; ERDEGs, Empagliflozin-Related Differentially Expressed Genes.

Supported by STRING, this study further constructed a PPI network for the screened 33 ERDEGs. As displayed in Fig 5A, 28 ERDEGs were interconnected, including IL1B, EGFR, ERBB2, JAK2, LGALS3, AGTR1, DPP4, MMP1, IGFBP3, SYK, PRF1, SLC2A1, MME, PDGFRB, LCK, ABCB1, SNCA, CDK1, BCL2A1, LGALS9, PYGM, HK2, MMP8, HCAR2, TBXA2R, PRKG1, PLK1, and PDE5A. Topological parameters for these ERDEGs were analyzed and are presented in Table 2. Subsequently, the top-10 ERDEGs were identified by employing the five algorithms (MCC, MNC, Degree, EPC, and Closeness) in the CytoHubba plugin of Cytoscape, with the results of calculation detailed in S2 Table. Simultaneously, corresponding PPI networks were visualized, with circle color changing from red to yellow to indicate decreasing scores (Fig 5B-F). The Venn diagram (Fig 5G) was also generated to identify the intersection of genes from the five algorithms, resulting in 6 hub genes, involving IL1B, EGFR, ERBB2, JAK2, SYK, and LGALS3 (S3 Table).

**Table 2:** Topological parameter analysis for connected ERDEGs.

Gene	MCC	MNC	Degree	EPC	Closeness
IL1B	248	17	19	16.654	22.667
EGFR	228	18	18	16.581	22.333
ERBB2	152	13	13	16.282	19.583
JAK2	102	8	8	15.277	17.167
LGALS3	40	8	8	14.742	16.917
AGTR1	15	6	7	13.758	16.833
DPP4	26	7	7	13.761	16.333
MMP1	60	6	6	14.193	15.917
IGFBP3	72	6	6	14.148	15.750
SYK	96	6	6	13.973	15.750
PRF1	50	6	6	13.869	15.750
SLC2A1	18	6	6	13.017	15.750
MME	18	5	5	12.810	15.333
PDGFRB	30	5	5	13.611	15.250
LCK	48	5	5	13.596	15.250
ABCB1	8	4	4	11.502	14.417
SNCA	6	3	3	10.991	14.250
CDK1	3	2	3	8.544	13.917
BCL2A1	2	2	2	8.732	13.750
LGALS9	4	3	3	10.102	13.667
PYGM	6	3	3	9.465	13.417
HK2	6	3	3	9.147	13.417
MMP8	1	1	1	5.982	12.500
HCAR2	1	1	1	5.677	12.500
TBXA2R	2	2	2	7.589	11.583
PRKG1	2	1	2	5.156	11.250
PLK1	1	1	1	3.268	9.450
PDE5A	1	1	1	2.319	8.200

ERDEGs, Empagliflozin-Related Differentially Expressed Genes; MCC, Maximal Clique Centrality; MNC, Maximum Neighborhood Component; EPC, Edge Percolated Component.

### 3.5. GO and KEGG pathway enrichment analyses of ERDEGs

**Fig 6:**
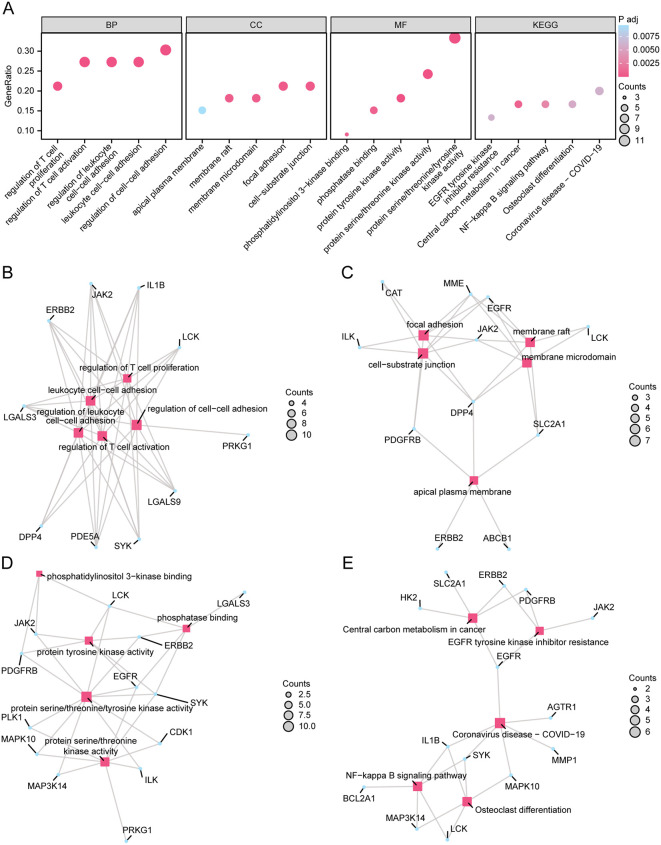
GO and KEGG Enrichment Analysis for ERDEGs. **A.** Bubble diagram of GO and KEGG enrichment analysis for ERDEGs: BP, CC, MF, and KEGG. GO terms and KEGG terms are shown on the abscissa. Bubble size represents gene count (number of genes enriched in each term), and bubble color intensity represents adjusted p-value (darker/more intense colors indicate higher significance). The x-axis shows gene ratio (proportion of genes enriched in each term). B-E. Network diagram of GO and KEGG enrichment analysis r for ERDEGs showing: BP **(B)**, CC **(C)**, MF **(D)**, and KEGG **(E)**. Pink nodes represent items, blue nodes represent molecules, and lines represent the relationship between items and molecules. ERDEGs, Empagliflozin-Related Differentially Expressed Genes; GO, Gene Ontology; KEGG, Kyoto Encyclopedia of Genes and Genomes; BP, Biological Process; CC, Cellular Component; MF, Molecular Function. The screening criteria for GO and KEGG enrichment analysis were adj.p < 0.05 and FDR value (q value) < 0.25, and the p value correction method was Benjamini-Hochberg (BH).

**Fig 7:**
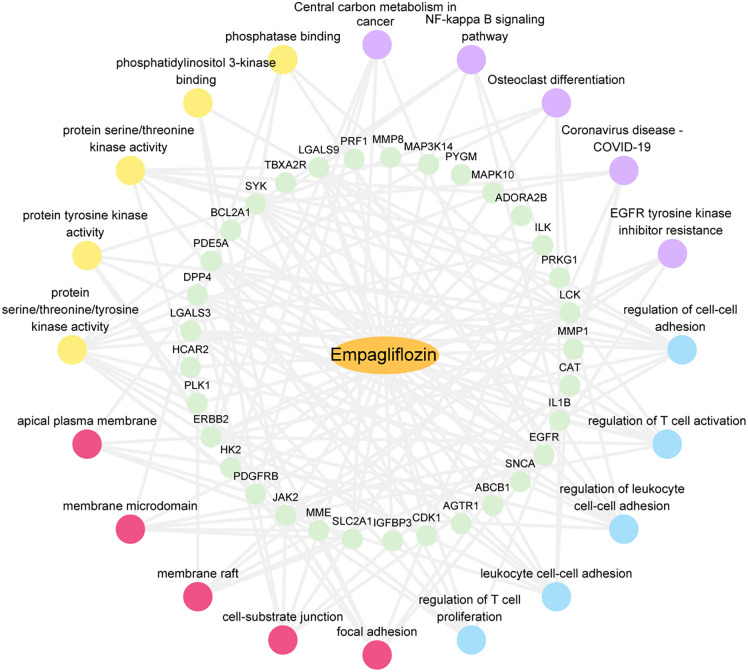
Drug-Targets-Pathways Network. Drug-target-pathway network diagram of empagliflozin, ERDEGs, GO, and KEGG. Orange ellipse is empagliflozin, green circles are ERDEGs, blue circles are biological processes, pink circles are cellular components, yellow circles are molecular functions, and purple circles are biological pathways (KEGG).

Our subsequent investigation focused on GO and KEGG pathway enrichment analyses of the 33 ERDEGs associated with AS (Table 3). Specifically, according to the GO enrichment analysis, the BPs mainly included regulation of cell-cell adhesion, regulation of T cell activation, regulation of leukocyte cell-cell adhesion, leukocyte cell-cell adhesion, and regulation of T cell proliferation; the cellular components (CCs) were mostly related to the focal adhesion, cell-substrate junction, membrane raft, membrane microdomain, and apical plasma membrane; and the molecular functions (MFs) were chiefly associated with protein serine/threonine/tyrosine kinase activity, protein tyrosine kinase activity, protein serine/threonine kinase activity, phosphatidylinositol 3-kinase binding, and phosphatase binding. Furthermore, as indicated by the KEGG enrichment analysis, the predominant pathways enriched were Central carbon metabolism in cancer, NF-kappa B signaling pathway, Osteoclast differentiation, Coronavirus disease-COVID-19, and EGFR tyrosine kinase inhibitor resistance. The results of the GO terms and KEGG pathways are visualized in the bubble chart (Fig 6A), while the network diagrams for each analysis are displayed in Fig 6B-E. Lastly, Fig 7 presents the overall drug-target-pathway network diagram, illustrating the interaction of empagliflozin, ERDEGs, GO terms, and enriched KEGG pathways.

**Table 3:** Results of GO and KEGG enrichment analysis for ERDEGs.

ONTOLOGY	ID	Description	GeneRatio	BgRatio	pvalue	p.adjust	qvalue
BP	GO:0022407	regulation of cell-cell adhesion	10/33	456/18800	3.59E-09	3.81E-06	1.90E-06
BP	GO:0050863	regulation of T cell activation	9/33	342/18800	5.16E-09	3.81E-06	1.90E-06
BP	GO:1903037	regulation of leukocyte cell-cell adhesion	9/33	344/18800	5.43E-09	3.81E-06	1.90E-06
BP	GO:0007159	leukocyte cell-cell adhesion	9/33	381/18800	1.32E-08	6.94E-06	3.46E-06
BP	GO:0042129	regulation of T cell proliferation	7/33	174/18800	1.80E-08	7.58E-06	3.78E-06
CC	GO:0005925	focal adhesion	7/33	419/19594	5.14E-06	4.61E-04	3.51E-04
CC	GO:0030055	cell-substrate junction	7/33	428/19594	5.91E-06	4.61E-04	3.51E-04
CC	GO:0045121	membrane raft	6/33	326/19594	1.54E-05	6.09E-04	4.65E-04
CC	GO:0098857	membrane microdomain	6/33	327/19594	1.56E-05	6.09E-04	4.65E-04
CC	GO:0016324	apical plasma membrane	5/33	358/19594	3.08E-04	9.62E-03	7.34E-03
MF	GO:0004712	protein serine/threonine/tyrosine kinase activity	11/33	446/18410	1.79E-10	3.36E-08	1.77E-08
MF	GO:0004713	protein tyrosine kinase activity	6/33	135/18410	1.31E-07	1.23E-05	6.48E-06
MF	GO:0004674	protein serine/threonine kinase activity	8/33	430/18410	6.90E-07	4.33E-05	2.28E-05
MF	GO:0043548	phosphatidylinositol 3-kinase binding	3/33	29/18410	1.86E-05	8.45E-04	4.45E-04
MF	GO:0019902	phosphatase binding	5/33	193/18410	2.25E-05	8.45E-04	4.45E-04
KEGG	hsa05230	Central carbon metabolism in cancer	5/30	70/8164	4.84E-06	8.81E-04	5.45E-04
KEGG	hsa04064	NF-kappa B signaling pathway	5/30	104/8164	3.37E-05	3.07E-03	1.90E-03
KEGG	hsa04380	Osteoclast differentiation	5/30	128/8164	9.11E-05	5.53E-03	3.42E-03
KEGG	hsa05171	Coronavirus disease - COVID-19	6/30	232/8164	1.65E-04	6.04E-03	3.73E-03
KEGG	hsa01521	EGFR tyrosine kinase inhibitor resistance	4/30	79/8164	1.84E-04	6.04E-03	3.73E-03

GO, Gene Ontology; BP, Biological Process; CC, Cell Components; MF, Molecular Function; KEGG, Kyoto Encyclopedia of Genes and Genomes; ERDEGs, Empagliflozin -Related Differentially Expressed Genes.

### 3.6. GSEA

**Fig 8:**
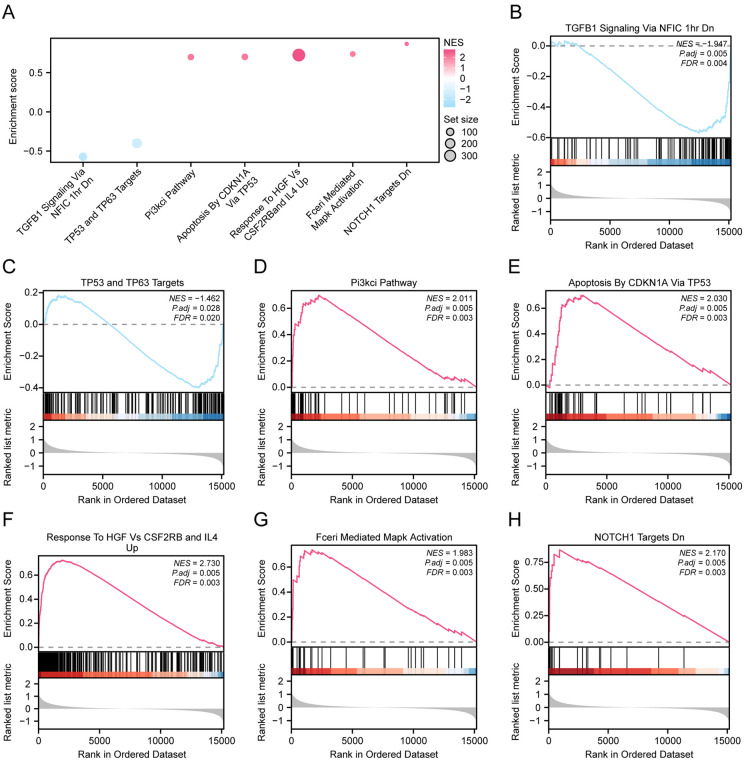
GSEA for Combined Dataset. **A.** Bubble plot showcasing the GSEA of 7 biological functions in the combined datasets. B-H. GSEA showed that all genes were significantly enriched in TGFB1 Signaling Via NFIC 1hr Dn **(B)**, TP53 And TP63 Targets **(C)**, Pi3kci Pathway **(D)**, NFIC 1HR DN **(B)**, TP53 and TP63 targets **(C)**, PI3KCI pathway **(D)**. Apoptosis By CDKN1A Via TP53 **(E)**, Response To HGF Vs CSF2RB And IL4 Up **(F)**, Fceri Mediated Mapk Activation **(G)**, NOTCH1 Targets Dn **(H)**. The enrichment score (ES) curve is shown at the top of each panel, with the ranking metric scores in the middle, and gene positions marked as vertical lines. NES: normalized enrichment score. Positive NES indicates enrichment in AS group; negative NES indicates enrichment in control group. GSEA, Gene Set Enrichment Analysis. The screening criteria of GSEA were adj.p < 0.05 and FDR value (q value) < 0.25, and the p value correction method was Benjamini-Hochberg (BH).

The impact of all gene expression levels associated with AS in the combined dataset were analyzed based on GSEA (Fig 8A and Table 4). All genes were significantly enriched in TGFB1 Signaling Via NFIC 1hr Dn (Fig 8B), TP53 And TP63 Targets (Fig 8C), Pi3kci Pathway (Fig 8D), Apoptosis By CDKN1A Via TP53 (Fig 8E), Response To HGF Vs CSF2RB And IL4 Up (Fig 8F), Fceri Mediated Mapk Activation (Fig 8G), and NOTCH1 Targets Dn (Fig 8H).

**Table 4:** Results of GSEA for combined datasets.

ID	Set Size	Enrichment Score	NES	p value	p.adjust	q value
MCLACHLAN_DENTAL_CARIES_UP	203	0.847196	3.022044	0.000322	0.004648	0.003332
POOLA_INVASIVE_BREAST_CANCER_UP	228	0.810295	2.933017	0.000316	0.004648	0.003332
BLANCO_MELO_COVID19_SARS_COV_2_POS_PATIENT_LUNG_TISSUE_UP	132	0.828531	2.810629	0.000332	0.004648	0.003332
RUTELLA_RESPONSE_TO_HGF_VS_CSF2RB_AND_IL4_UP	351	0.724795	2.729908	0.000302	0.004648	0.003332
FULCHER_INFLAMMATORY_RESPONSE_LECTIN_VS_LPS_DN	369	0.713356	2.702787	0.000298	0.004648	0.003332
FLECHNER_BIOPSY_KIDNEY_TRANSPLANT_REJECTED_VS_OK_UP	75	0.858885	2.695737	0.000346	0.004648	0.003332
VILIMAS_NOTCH1_TARGETS_DN	23	0.867384	2.170452	0.000375	0.004648	0.003332
WU_APOPTOSIS_BY_CDKN1A_VIA_TP53	47	0.701504	2.030310	0.000356	0.004648	0.003332
PID_PI3KCI_PATHWAY	46	0.698996	2.010575	0.000360	0.004648	0.003332
REACTOME_FCERI_MEDIATED_MAPK_ACTIVATION	32	0.737948	1.982604	0.000366	0.004648	0.003332
PEREZ_TP53_AND_TP63_TARGETS	167	−0.400080	−1.462285	0.004685	0.027603	0.019787
PLASARI_TGFB1_SIGNALING_VIA_NFIC_1HR_DN	97	−0.571784	−1.946640	0.000473	0.005279	0.003785
MCLACHLAN_DENTAL_CARIES_DN	69	−0.685154	−2.198413	0.000464	0.005279	0.003785
DELYS_THYROID_CANCER_DN	205	−0.585770	−2.199327	0.000528	0.005631	0.004036
VECCHI_GASTRIC_CANCER_EARLY_DN	300	−0.563971	−2.208162	0.000558	0.005773	0.004138
TOMLINS_PROSTATE_CANCER_DN	35	−0.777394	−2.220623	0.000435	0.005146	0.003689
RICKMAN_HEAD_AND_NECK_CANCER_F	51	−0.754670	−2.294535	0.000456	0.005279	0.003785
BERTUCCI_MEDULLARY_VS_DUCTAL_BREAST_CANCER_DN	143	−0.684095	−2.455397	0.000508	0.005551	0.003979
LIU_PROSTATE_CANCER_DN	412	−0.636094	−2.558721	0.000616	0.006110	0.004380
PAPASPYRIDONOS_UNSTABLE_ATEROSCLEROTIC_PLAQUE_DN	37	−0.923506	−2.657400	0.000444	0.005218	0.003741

GSEA, Gene Set Enrichment Analysis.

### 3.7. GSVA

**Fig 9:**
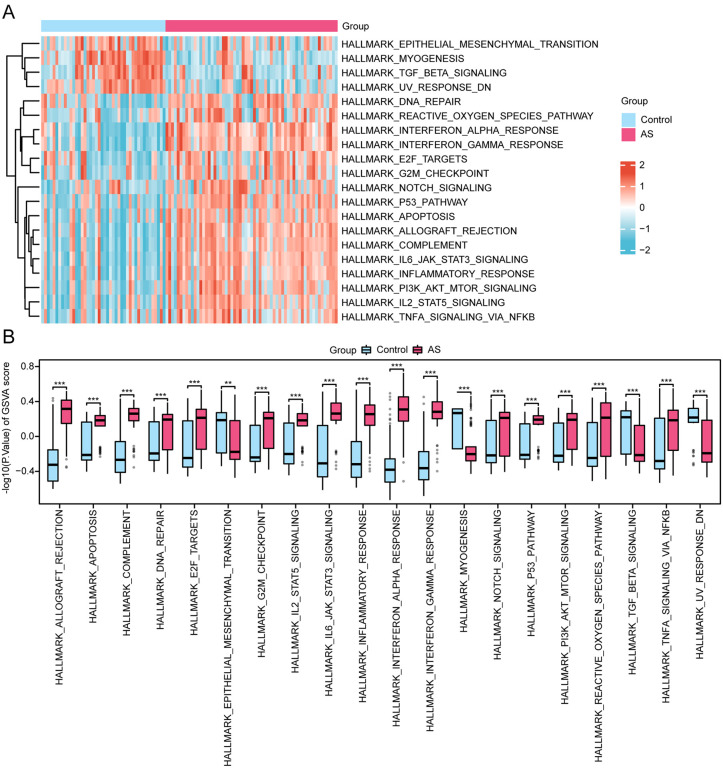
GSVA Analysis for Combined Dataset. A-B. Heatmap (A) and group comparison plot (B) of GSVA results between AS and controls of the combined dataset. AS, Atherosclerosis; GSVA, Gene Set Variation Analysis. ** represents p value < 0.01, highly statistically significant; *** represents p value < 0.001 and highly statistically significant. Pink represents AS and blue represents Control. Blue represents down-regulation and red represents up-regulation in the heat map. The screening criteria for GSVA was adj.p < 0.05, and the p value correction method was Benjamini-Hochberg (BH).

Our research further compared the pathway differences in genes involved in AS and controls from the MSigDB database (Table 5). The top-20 enriched pathways (an adj. p < 0.05 and |logFC|) were identified and are visualized in the heatmap (Fig 9A). Through Mann–Whitney U test, the inter-group differencesare illustrated in Fig 9B. Consequently, there were statistically significant differences in pathways such as HALLMARK_MYOGENESIS, HALLMARK_UV_RESPONSE_DN, HALLMARK_TGF_BETA_SIGNALING, HALLMARK_EPITHELIAL_MESENCHYMAL_TRANSITION, HALLMARK_DNA_REPAIR, HALLMARK_NOTCH_SIGNALING, HALLMARK_APOPTOSIS, HALLMARK_G2M_CHECKPOINT, HALLMARK_REACTIVE_OXYGEN_SPECIES_PATHWAY, HALLMARK_TNFA_SIGNALING_VIA_NFKB, HALLMARK_IL2_STAT5_SIGNALING, HALLMARK_E2F_TARGETS, HALLMARK_PI3K_AKT_MTOR_SIGNALING, HALLMARK_P53_PATHWAY, HALLMARK_COMPLEMENT, HALLMARK_INFLAMMATORY_RESPONSE, HALLMARK_IL6_JAK_STAT3_SIGNALING, HALLMARK_ALLOGRAFT_REJECTION, HALLMARK_INTERFERON_GAMMA_RESPONSE, and HALLMARK_INTERFERON_ALPHA_RESPONSE, between the AS group and the control group (all p < 0.05).

**Table 5:** Results of GSVA for combined datasets.

ID	logFC	AveExpr	p value	adj.p value
HALLMARK_MYOGENESIS	0.291286	−0.020213	2.27E-09	1.62E-08
HALLMARK_UV_RESPONSE_DN	0.262392	0.009072	7.58E-08	3.66E-07
HALLMARK_TGF_BETA_SIGNALING	0.215072	−0.030827	1.75E-05	4.85E-05
HALLMARK_EPITHELIAL_MESENCHYMAL_TRANSITION	0.152181	0.005497	2.80E-03	5.00E-03
HALLMARK_DNA_REPAIR	−0.215413	0.021013	3.03E-06	1.08E-05
HALLMARK_NOTCH_SIGNALING	−0.230315	0.009052	6.06E-06	1.89E-05
HALLMARK_APOPTOSIS	−0.233294	0.024750	8.06E-08	3.66E-07
HALLMARK_G2M_CHECKPOINT	−0.235461	0.008687	2.26E-06	8.70E-06
HALLMARK_REACTIVE_OXYGEN_SPECIES_PATHWAY	−0.238394	0.002213	4.72E-05	1.06E-04
HALLMARK_TNFA_SIGNALING_VIA_NFKB	−0.238602	−0.001967	4.89E-05	1.06E-04
HALLMARK_IL2_STAT5_SIGNALING	−0.240823	0.028500	2.39E-07	9.94E-07
HALLMARK_E2F_TARGETS	−0.245382	0.015494	5.48E-06	1.83E-05
HALLMARK_PI3K_AKT_MTOR_SIGNALING	−0.248379	0.015165	5.53E-08	3.07E-07
HALLMARK_P53_PATHWAY	−0.249181	0.021029	2.91E-09	1.82E-08
HALLMARK_COMPLEMENT	−0.388038	0.034139	5.48E-14	5.48E-13
HALLMARK_INFLAMMATORY_RESPONSE	−0.398947	0.029156	9.14E-12	7.62E-11
HALLMARK_IL6_JAK_STAT3_SIGNALING	−0.446064	0.046452	3.17E-14	3.97E-13
HALLMARK_ALLOGRAFT_REJECTION	−0.463820	0.030452	9.69E-15	1.62E-13
HALLMARK_INTERFERON_GAMMA_RESPONSE	−0.518896	0.047431	7.70E-18	1.92E-16
HALLMARK_INTERFERON_ALPHA_RESPONSE	−0.582619	0.045021	6.25E-19	3.12E-17

GSVA, Gene Set Variation Analysis.

### 3.8. Regulatory network of hub genes

**Fig 10:**
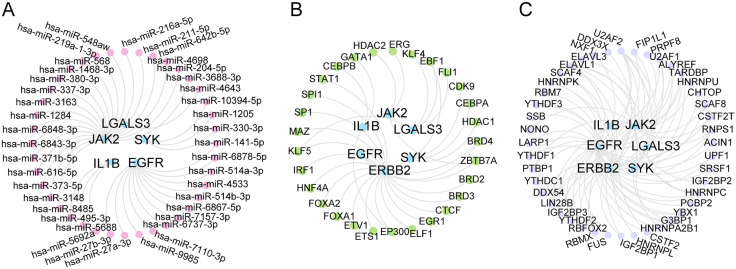
Regulatory Network of Hub Genes. A. mRNA-miRNA Regulatory Network of empagliflozin-related Hub Genes. B. mRNA-TF Regulatory Network of empagliflozin-related Hub Genes. C. mRNA-RBP Regulatory Network of empagliflozin-related Hub Genes. E; Empagliflozin; TF, Transcription Factor; RBP, RNA-Binding Protein. mRNAs are shown in blue, miRNAs in pink, TFs in green, and RBP in purple.

Following the analysis of data sourced from miRDB database, the mRNA-miRNA regulatory network (Fig 10A and S4 Table) revealed 5 hub genes and 40 miRNAs. Furthermore, the integration of 6 hub genes and 29 TFs from the ChIPBase and hTFtarget generated a mRNA-TF regulatory network, as presented in Fig 10B and S5 Table. Additionally, 44 RBPs predicted to bind to the 6 hub genes were identified using the StarBase database (Fig 10C and S6 Table).

### 3.9. Correlation and Friends analysis among empagliflozin-related hub genes

**Fig 11:**
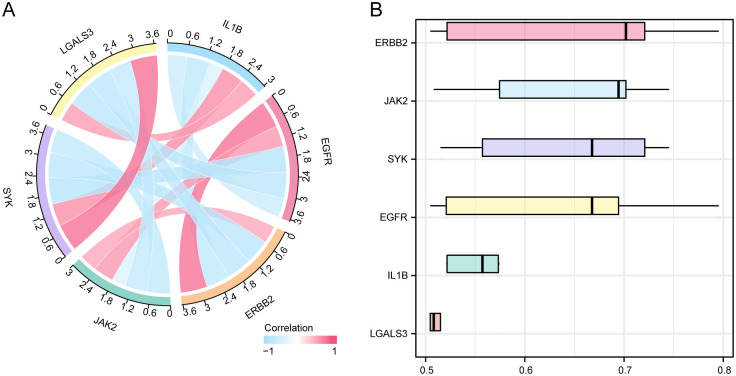
Correlation and Friends Analysis of Hub Genes. **A.** Chord diagram of the correlations among the 6 empagliflozin-related hub genes in the combined datasets **B.** Box plot of functional similarity (Friends) analysis results for hub genes. Pink represents a positive correlation, blue represents a negative correlation. The absolute value of the correlation coefficient (r value) below 0.3 is weak or no correlation, between 0.3 and 0.5 is weak correlation, between 0.5 and 0.8 is moderate correlation, and above 0.8 is strong correlation.

As illustrated in Fig 11A, the Chord diagram was employed to visualize the results from pairwise correlations of the 6 hub genes identified by Pearson correlation analysis, with negative correlations found in these hub genes primarily. Specifically, IL1B, SYK, and LGALS3 were positively correlated with each other but negatively correlated with ERBB2, EGFR, and JAK2, which conversely showed positive correlations. Additionally, ERBB2 was found to play an important role in AS, as evidenced by the box plot of functional similarity (Friends) analysis (Fig 11B).

### 3.10. ROC curve analysis of the correlation between hub gene expression and AS clinical characteristics

**Fig 12:**
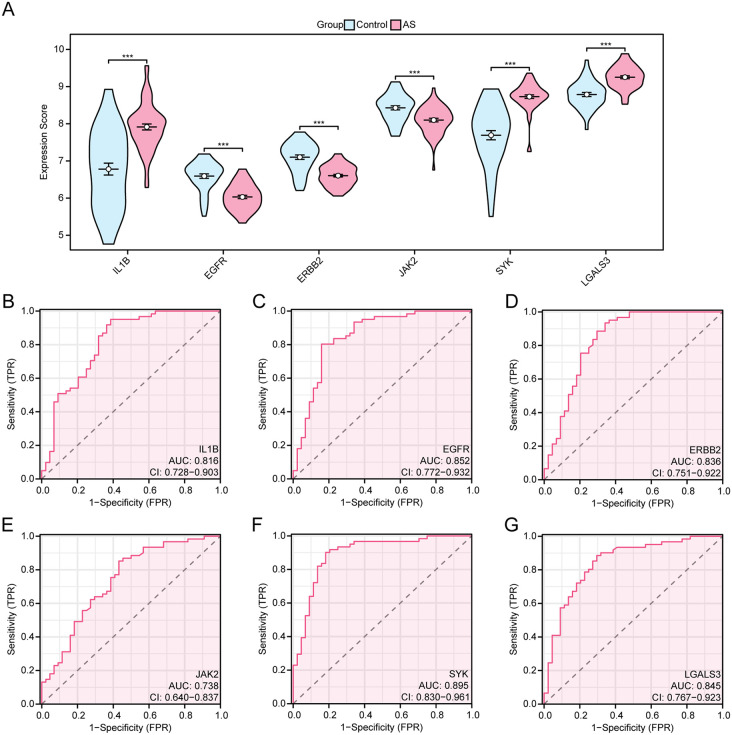
Differential Expression Analysis and ROC Curve Analysis. **A.** Comparison plot of hub genes in AS and control groups within the combined dataset. B-G. ROC curves of hub genes in the combined dataset. *** represents a p value < 0.001 and highly statistically significant. When AUC > 0.5, it indicates that the expression of the molecule is a trend to promote the occurrence of the event, and the closer the AUC is to 1, the better the diagnostic effect. The AUC had some accuracy in the range of 0.7 to 0.9. AS, Atherosclerosis; ROC, Receiver Operating Characteristic; AUC, Area Under the Curve. TPR, True Positive Rate; FPR, False Positive Rate. Blue represents Control and pink represents AS.

In the combined dataset, inter-group differences were observed in expression levels of IL1B, EGFR, ERBB2, JAK2, SYK, and LGALS3 between the AS and control groups (Fig 12A). As a result, there were highly statistically significant differences in the expression levels of the 6 hub genes between groups(all p 0.001). Among these, IL1B, SYK and LGALS3 showed significantly up-regulated expressions, while EGFR, ERBB2, and JAK2 exhibited obviously down-regulated expressions. Subsequently, based on the ROC curve analysis, the expression levels of IL1B, EGFR, ERBB2, JAK2, SYK, and LGALS3 exhibited reasonable accuracy in distinguishing AS and the controls, with corresponding AUC values of 0.816, 0.852, 0.836, 0.738, 0.895, and 0.845, respectively (Fig 12B-G).

### 3.11. Validation of hub genes

**Fig 13:**
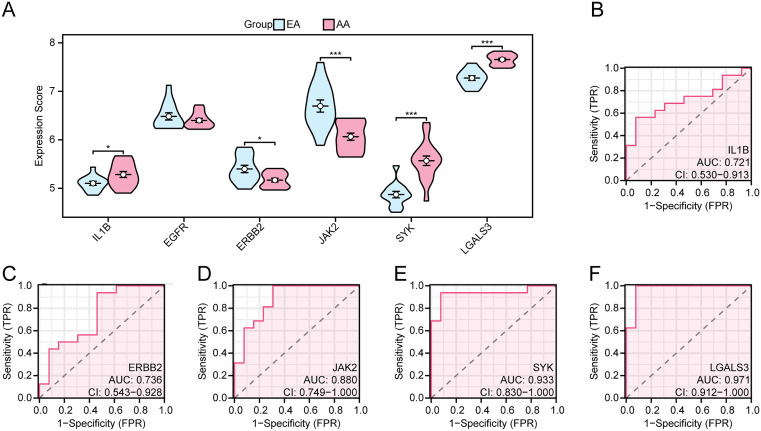
Differential Expression Validation and ROC Curve Analysis. **A.** Comparison plot of hub genes in AA samples and EA samples within the validation set GSE28829. B-F. ROC curve of hub genes in the validation set GSE28829. *** represents p value < 0.001, highly statistically significant; * represents p value < 0.05, indicating statistical significance. When AUC > 0.5, it indicates that the expression of the molecule is a trend to promote the occurrence of the event, and the closer the AUC is to 1, the better the diagnostic effect. AUC between 0.7-0.9 has a certain accuracy; AUC > 0.9 has high accuracy. AA, Advanced Atherosclerotic Plaque Samples; EA, Early Atherosclerotic Plaque Samples; ROC, Receiver Operating Characteristic; AUC, Area Under the Curve. TPR, True Positive Rate; FPR, False Positive Rate. Pink represents AA samples and blue represents early EA samples.

In accordance with the analysis of the hub gene expression in the validation set GSE28829, five of the 6 hub genes (i.e., JAK2, SYK, LGALS3, IL1B, and ERBB2) revealed statistically significant differences in expression levels between EA and AA groups, exhibiting high consistency with the findings from the combined dataset (Fig 13A). Specifically, compared to EA group, IL1B, SYK, and LGALS3 showed higher expression, while ERBB2 and JAK2 exhibited lower expression levels in the AA group. Moreover, the expression levels of IL1B, ERBB2, JAK2, SYK, and LGALS3 could accurately distinguish between EA and AA groups, as depicted in the the ROC curve, with AUC values of 0.721, 0.736, 0.880, 0.933, and 0.971, respectively (Fig 13B-F).

### 3.12. Immune cell characteristics and hub gene correlation analysis

**Fig 14:**
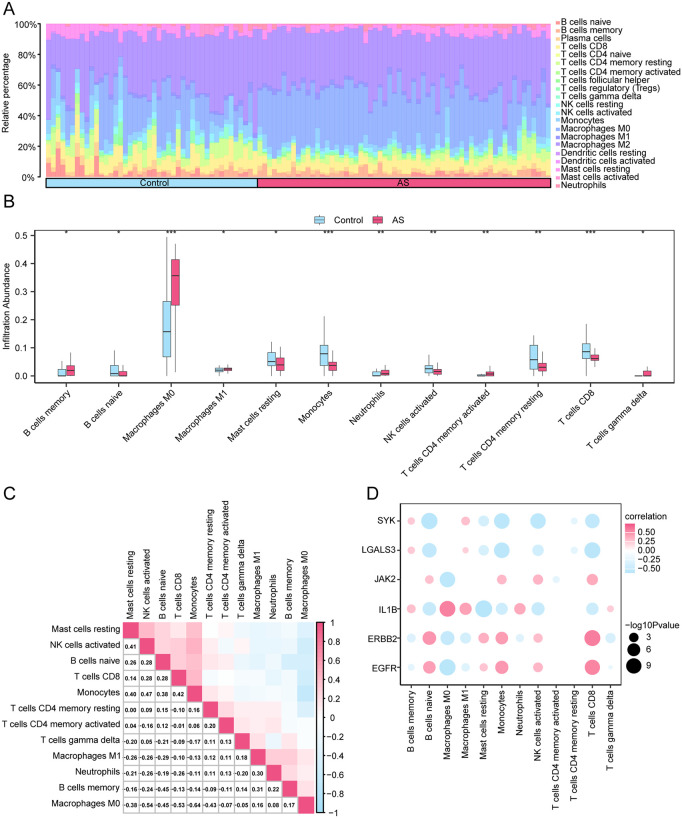
Combined Datasets Immune Infiltration Analysis by CIBERSORT Algorithm. A-B. Bar graph (A) and group comparison graph (B) of the proportion of immune cells in the combined dataset. **C.** Correlation heatmap of immune cell infiltration abundance in the combined dataset. **D.** Bubble plot of the correlation between hub genes and immune cell infiltration abundance in the combined dataset. AS, Atherosclerosis. * represents p value < 0.05, indicating statistical significance; ** represents p value < 0.01, highly statistically significant; *** represents p value < 0.001 and highly statistically significant. The absolute value of the correlation coefficient (r-value) below 0.3 was weak or no correlation, between 0.3 and 0.5 was a weak correlation, between 0.5 and 0.8 was a moderate correlation, and above 0.8 was a strong correlation. Pink is AS and blue is Control. Blue is a negative correlation, pink is a positive correlation, and the depth of the color represents the strength of the correlation.

This study observed remarkable correlations between immune cells and empagliflozin-related hub genes, when adopting CIBERSORT analysis and ssGSEA algorithm. To be specific, shown in Fig 14A. Inter-group comparisons showed significant differences in the proportion of immune cell infiltration between AS and control groups. Specifically, statistically significant differences in expressions were found in 12 immune cell types (all p < 0.05), including B cells naive, B cells memory, T cells CD8, T cells CD4 memory resting, T cells CD4 memory activated, T cells gamma delta, NK cells activated, monocytes, macrophages M0, macrophages M1, mast cells resting, and neutrophils (Fig 14B). The correlation thermogram in Fig 14C highlighted strong positive correlation between monocytes and NK cells activated (r = 0.47), as well as the greatest negative correlation between macrophages M0 and monocytes (r = −0.64). Fig 14D shows the correlation between 6 hub genes and the 12 immune cell types infiltration. Consequently, ERBB2 was significantly positively correlated with T cells CD8 (r = 0.58, p < 0.05), while IL1B was highly negatively correlated with mast cells resting (r = −0.61, p < 0.05).

**Fig 15:**
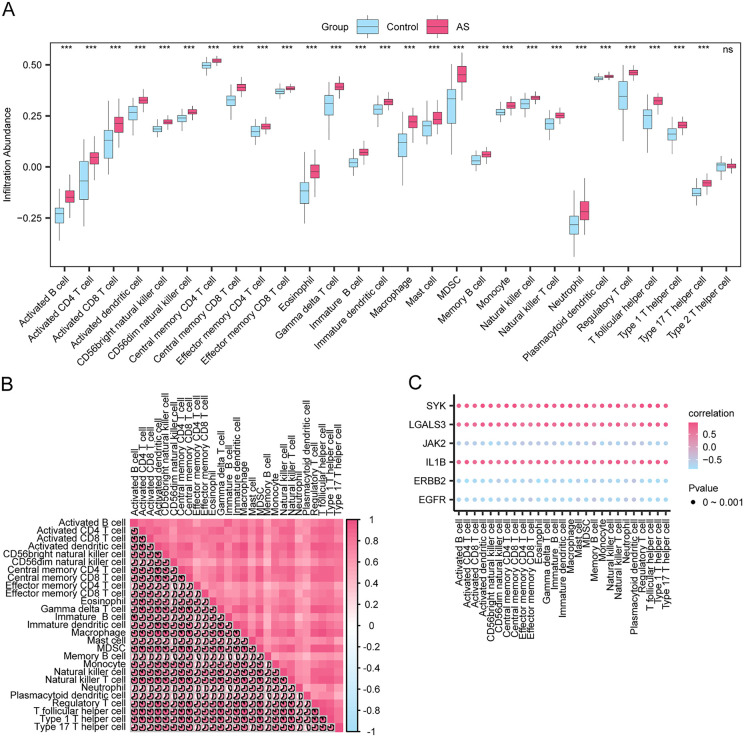
Immune Infiltration Analysis by ssGSEA Algorithm. **A.** Group comparison plots of immune cells in AS and Control of the combined dataset. **B.** Correlation heatmap of immune cell infiltration abundance in the combined dataset. **C.** Bubble plot of the correlation between hub genes and immune cell infiltration abundance in the combined dataset. ssGSEA, single-sample Gene-Set Enrichment Analysis; AS, Atherosclerosis. ns stands for p value ≥ 0.05, not statistically significant; *** represents p value < 0.001, highly statistically significant. The absolute value of the correlation coefficient (r-value) below 0.3 was weak or no correlation, between 0.3 and 0.5 was a weak correlation, between 0.5 and 0.8 was a moderate correlation, and above 0.8 was a strong correlation. Pink is AS and blue is Control. Pink is a positive correlation and blue is a negative correlation. The depth of the color represents the strength of the correlation.

In view of the calculation results via the ssGSEA algorithm, this study also confrmed the characterized immune subtypes based on the abundance of 28 immune cells (Fig 15A). The correlation thermogram indicated a positive relationship between these immune cell types (Fig 15B). In the bubble plot (Fig 15C), a strongest positive correlation was found between LGALS3 and eosinophils (r = 0.70, p < 0.05), while a strongest negative correlation between JAK2 and CD56dim natural killer cells (r = −0.69, p < 0.05).

## 4. Discussion

### 4.1. Main findings

From the most comprehensive perspective to date, the present study analyzed the genetic and molecular mechanisms underlying SGLT2 inhibitors in AS, and investigated into their interactions within the human genome. With the identification of 33 genes as ERDEGs, six genes (IL1B, EGFR, ERBB2, JAK2, SYK, and LGALS3) were identified as hub genes, with the discovery of interactions with miRNAs, TFs, and RBPs, as suggested by a regulatory network. Furthermore, it was observed with significant inter-group differences in the expression levels of the hub genes between AS and control groups, including up-regulated IL1B, SYK, and LGALS3, as well as down-regulated EGFR, ERBB2, and JAK2. Meanwhile, ROC curve demonstrated the accuracy of the hub genes in distinguishing these two groups, with subsequent verification in the validation set. In addition, the identified hub genes were also found to have potential relationships with immune cell subsets, with ERBB2 exhibiting significant positive correlation with T cells CD8, IL1B showing significant negative correlation with mast cells resting, LGALS3 having the strongest positive correlation with eosinophils, and JAK2 revealing the strongest negative correlation with CD56dim natural killer cells.

### 4.2. GO and KEGG analyses on ERDEGs

GO and KEGG analyses were conducted on ERDEGs to elucidate their roles in biological processes and metabolic pathways. Specifically, as indicated by GO analysis, ERDEGs were primarily involved in PBs such as the regulation of leukocyte cell-cell adhesion, leukocyte cell-cell adhesion, and regulation of T cell proliferation; significant enrichment regarding MFs in various enzyme activities and protein interactions. These results underscore the crucial roles of these biological processes and activities in multiple stages of AS, such as endothelial cell damage and dysfunction, smooth muscle cell proliferation and migration, inflammatory cell recruitment and activation, as well as plaque formation and stabilization [47–51].

Meanwhile, as revealed by the KEGG pathway analysis, the screened genes were mainly involved in central carbon metabolism in cancer, NF-kappa B signaling pathway, osteoclast differentiation, COVID-19, and EGFR tyrosine kinase inhibitor resistance, all of which are important in AS development and progression [47,52,53]. Notably, the NF-kappa B signaling pathway plays a central role in inflammation and immunity [54].

Collectively, empagliflozin is critical in modulating AS-associated signaling pathways and gene expression, potentially explaining its mechanisms against the disease, which may facilitate the identification of new strategies to treat AS.

### 4.3. Characteristics of the six empagliflozin-related hub genes in AS

Through network pharmacology and bioinformatics-based approach, our study identified 6 hub genes that are dysregulated in AS and potentially associated with the anti-atherosclerotic effect of empagliflozin. The proteins encoded by these genes are primarily involved in inflammatory reaction, a significant event in AS occurrence and development. Specifically, IL1B, SYK, and LGALS3 participate significantly in the process of inflammatory reactions; [55–57] moreover, JAK2, EGFR, ERBB2, and IL1B are contributors to cell proliferation and migration, which are also essential processes in AS where smooth muscle cells undergo abnormal migration and vascular remodeling [58–60]. Given these findings, it may offer promising solutions to treat various diseases, including AS, by targeting these proteins with small molecule inhibitors and antibody drug. For instance, the CANTOS trial demonstrated reduced recurrent major adverse cardiovascular events by using anti-IL-1β antibody in treating stable cardiovascular patients post-myocardial infarction, along with standard care [61,62].

IL1B encodes interleukin-1β, a master regulator of inflammatory responses in atherosclerosis. Empagliflozin reduces IL-1B secretion through NLRP3 inflammasome inhibition and targets the TRAF3IP2/ROS/NLRP3/Caspase-1 pathway [63]. SGLT2 inhibitors significantly reduce cardiac inflammatory cytokine expression, including IL-1B, suggesting reduced inflammation-mediated vascular damage [64]. The anti-atherosclerotic effects are mediated through β-hydroxybutyrate-dependent mechanisms that suppress inflammasome activation.

SYK is a critical mediator involved in atherosclerosis progression through inflammatory signaling pathways. SYK inhibition demonstrates significant anti-proliferative effects on vascular smooth muscle cells, attenuating cell proliferation and migration associated with atherosclerosis development. SYK serves as a key mediator of inflammation in atherosclerotic processes, particularly in PCSK9-induced inflammatory responses [65]. Empagliflozin’s cardiovascular protective effects may involve SYK pathway modulation, contributing to reduced vascular inflammation and improved plaque stability.

LGALS3 encodes Galectin-3, a multifunctional protein involved in atherosclerosis progression through macrophage activation and foam cell formation. The upregulation of LGALS3 in atherosclerotic lesions suggests its involvement in inflammatory cascades, though its exact role warrants further investigation. Empagliflozin may modulate Gal-3 expression through anti-inflammatory pathways.

EGFR, a member of the ErbB receptor tyrosine kinase family, plays a critical role in atherosclerosis development. Myeloid cell-specific deletion of EGFR significantly reduces atherosclerotic lesion size by 43−54% through decreased macrophage accumulation, TNF-αand IL-6 production, and limited lipid uptake via CD36 downregulation [66]. ERBB2 (HER2) is abundantly expressed in endothelial cells and vascular smooth muscle cells, with elevated serum HER2 levels independently associated with coronary artery disease presence and stenotic vessel number. The downregulation of EGFR and ERBB2 observed in our analysis suggests empagliflozin may inhibit vascular smooth muscle cell proliferation and inflammatory responses, contributing to its anti-atherosclerotic effects.

JAK2 encodes Janus kinase 2, a critical tyrosine kinase mediating cytokine signaling through the JAK-STAT pathway. The JAK2V617F mutation confers a 12-fold increased cardiovascular disease risk, promoting accelerated atherosclerosis with increased inflammation, larger necrotic cores, and defective efferocytosis [67]. However, normal macrophage JAK2 is atheroprotective, as its deficiency causes impaired cholesterol efflux and accelerated atherosclerosis [68]. The JAK2/STAT3 pathway drives M1 macrophage polarization and NLRP3 inflammasome activation. Empagliflozin downregulates the JAK2/STAT1 pathway in macrophages, reducing inflammatory responses, suggesting empagliflozin’s cardioprotective effects partially involve JAK2 pathway modulation.

### 4.4. Critical assessment of findings

While our network pharmacology approach identified six hub genes with statistical significance, several limitations warrant careful interpretation. First, the modest fold change threshold (|logFC| ≥ 0.25) employed in this study, though justified for pathway analysis, means that individual gene expression changes may be biologically subtle. The clinical relevance of such modest expression differences remains uncertain without dose-response validation or functional studies demonstrating that these changes translate to meaningful biological effects. Second, the correlations identified between hub genes and immune cell subsets, while statistically significant, represent associations rather than causal relationships. For instance, the correlation between ERBB2 and CD8 + T cells (r = 0.58) or LGALS3 and eosinophils (r = 0.70) does not establish whether these genes directly regulate immune cell infiltration or merely reflect parallel processes in atherosclerotic tissue. Third, our findings are derived from cross-sectional gene expression data and cannot determine whether the observed dysregulation of these hub genes precedes, accompanies, or follows empagliflozin’s therapeutic effects. Given that empagliflozin’s cardiovascular benefits occur through multiple mechanisms (metabolic, hemodynamic, and anti-inflammatory), the relative contribution of each identified hub gene to its overall anti-atherosclerotic effect remains speculative without targeted intervention studies.

### 4.5. Immune cell characteristics and hub gene correlation analysis

Based on CIBERSORT and ssGSEA analyses, there were significant differences in the proportions and infiltration levels of immune cells between AS and controls. Our subsequent analyses also confirmed the relationships between hub genes and immune cell subsets, with significant correlations between ERBB2 and CD8 + T cells, IL1B and resting mast cells, LGALS3 and eosinophils, and JAK2 and CD56dim NK cells. Collectively, we may acquire novel perspective on the role of empagliflozin in immune regulation for anti-atherosclerotic effects, and additional targets for developing novel immunotherapeutic strategies. For further in-depth understanding, our future study should continue to explore the relationship between these immune cells and disease progression as well as treatment response.

### 4.6. Clinical importance

Our study has significant implications for both clinical practice and future research. First, our study may offer important scientific basis for future clinical research and treatment strategies, as evidenced by a comprehensive explanation of the mechanism of empagliflozin against AS. In particular, this study reveals significant correlations of empagliflozin-related hub genes with immune cell subsets, which are key players in AS onset and progression [69]. All these findings may hint future directions of validation and exploration of potential applications of empagliflozin clinically.

### 4.7. Strengths and limitations

Our research has significant advantages. Firstly, this study comprehensively explored the potential mechanisms of empagliflozin against AS within the human genome utilizing advanced network pharmacology methods, robust public data platforms, and powerful analytical tools. Secondly, our research addressed the limitations of animal experimental studies, which often fail to fully replicate human conditions that may hinder drug discovery and clinical translation.

However, the existence of several limitations underlines our cautious interpretation of results in the current study. Firstly, study based on network pharmacology, constrained by the timeliness of public databases, may be insufficient to realize real-time investigations owing to the potentially outdated target information. Secondly, this study did not validate the results of network pharmacology, necessitating more tangible pharmacological models, as reliance solely on computer and database networks may not be sufficient. Third, this study lacks experimental validation through wet lab or in vivo experiments, which limits the direct clinical translation of our findings. The identified hub genes and pathways require validation through cell culture studies, animal models, and ultimately clinical trials to confirm their therapeutic relevance. Moreover, the immune infiltration analysis using CIBERSORT and ssGSEA provides computational estimates rather than direct measurements of immune cell proportions. These deconvolution methods depend on reference signatures that may not fully represent immune cells in diseased atherosclerotic tissues, and bulk RNA sequencing has inherent limitations in distinguishing closely related cell subtypes. Additionally, different deconvolution algorithms can yield variable estimates for the same samples, and spatial information about immune cell distribution within plaques cannot be captured. Future validation using single-cell RNA sequencing, flow cytometry, or multiplexed immunofluorescence would provide more accurate immune cell characterization and confirm these computational predictions.

Therefore, our findings should be validated by further *in vitro* experiments and clinical trials, thereby promoting the clinical application of the discovered therapeutic targets.

## 5. Conclusion

In conclusion, this study represents the first comprehensive integration of network pharmacology and immune infiltration analysis to elucidate empagliflozin’s anti-atherosclerotic mechanisms. Our identification of six key hub genes (IL1B, EGFR, ERBB2, JAK2, SYK, and LGALS3) and their correlations with immune cell subsets provides a molecular framework for understanding empagliflozin’s cardiovascular protective effects. These findings lay the groundwork for future experimental validation studies and suggest potential combination therapeutic strategies targeting both metabolic and inflammatory pathways in atherosclerosis treatment.

## Supporting information

S1 Table Empagliflozin-Related Genes (ERGs) in Atherosclerosis. Complete list of 76 genes identified at the intersection of empagliflozin targets and atherosclerosis-related genes from PubChem, SwissTargetPrediction, DGIdb, CTD, and GeneCards databases.
(XLSX)

S2 Table CytoHubba Algorithm Results for ERDEGs. Top-10 genes ranked by five topological algorithms (MCC, MNC, Degree, EPC, and Closeness) from the CytoHubba plugin analysis of the protein-protein interaction network.
(XLSX)

S3 Table Empagliflozin-Related Hub Genes in Atherosclerosis. List of six hub genes (IL1B, EGFR, ERBB2, JAK2, SYK, and LGALS3) identified at the intersection of the five CytoHubba algorithms.
(XLSX)

S4 Table mRNA-miRNA Regulatory Network. Complete list of predicted interactions between 5 empagliflozin-related hub genes and 40 microRNAs identified from the miRDB database.
(XLSX)

S5 Table mRNA-Transcription Factor Regulatory Network Complete list of predicted interactions between 6 empagliflozin-related hub genes and 29 transcription factors identified from ChIPBase and hTFtarget databases.
(XLSX)

S6 Table mRNA-RNA Binding Protein Regulatory Network Complete list of predicted interactions between 6 empagliflozin-related hub genes and 44 RNA-binding proteins identified from the starBase v3.0 database.
(XLSX)
